# Assessing the utility of conserving evolutionary history

**DOI:** 10.1111/brv.12526

**Published:** 2019-05-31

**Authors:** Caroline M. Tucker, Tracy Aze, Marc W. Cadotte, Juan L. Cantalapiedra, Chelsea Chisholm, Sandra Díaz, Richard Grenyer, Danwei Huang, Florent Mazel, William D. Pearse, Matthew W. Pennell, Marten Winter, Arne O. Mooers

**Affiliations:** ^1^ Department of Biology University of North Carolina at Chapel Hill, Coker Hall, CB #3280 120 South Road Chapel Hill, NC 27599‐3280 U.S.A.; ^2^ Centre d'Écologie Fonctionnelle et Évolutive (UMR 5175), CNRS 34090 Montpellier France; ^3^ School of Earth and Environment, Maths/Earth and Environment Building University of Leeds Leeds LS2 9JT U.K.; ^4^ Department of Biological Sciences University of Toronto Scarborough, 1265 Military Trail Toronto ON M1C 1A4 Canada; ^5^ Department of Ecology and Evolutionary Biology University of Toronto, 25 Willcocks Street Toronto ON M5S 3B2 Canada; ^6^ Museum für Naturkunde, Leibniz‐Institut für Evolutions und Biodiversitätsforschung, Invalidenstraße 43 10115 Berlin Germany; ^7^ Departamento de Ciencias de la Vida Universidad de Alcalá 28805 Alcalá de Henares Madrid Spain; ^8^ Department of Ecology and Evolution Quartier UNIL‐Sorge Batiment Biophore CH‐1015 Lausanne Switzerland; ^9^ Instituto Multidisciplinario de Biología Vegetal (IMBIV), Consejo Nacional de Investigaciones Científicas y Técnicas and Facultad de Ciencias Exactas, Físicas y Naturales Universidad Nacional de Córdoba, Casilla de Correo 495 5000 Córdoba Argentina; ^10^ School of Geography and the Environment South Parks Road, University of Oxford Oxford OX1 3QY U.K.; ^11^ Department of Biological Sciences and Tropical Marine Science Institute National University of Singapore, 16 Science Drive 4, 117558 Singapore; ^12^ Department of Biological Sciences 8888 University Drive, Simon Fraser University Burnaby BC V5A 1S6, Canada; ^13^ Department of Botany 2329 West Mall, University of British Columbia Vancouver BC V6T 1Z4 Canada; ^14^ Biodiversity Research Centre 2212 Main Mall, University of British Columbia Vancouver BC V6T 1Z4 Canada; ^15^ Department of Biology & Ecology Center 5205 Old Main Hill, Utah State University Logan UT 84322, U.S.A.; ^16^ Department of Zoology South Parks Road, University of British Columbia Vancouver BC V6T 1Z4 Canada; ^17^ German Centre for Integrative Biodiversity Research (iDiv) Deutscher Platz 5E, 04103 Leipzig Germany

**Keywords:** phylogenetic diversity, conservation, prioritization, phenotypic diversity, ecosystem function, extinction, functional diversity, benefits to people

## Abstract

It is often claimed that conserving evolutionary history is more efficient than species‐based approaches for capturing the attributes of biodiversity that benefit people. This claim underpins academic analyses and recommendations about the distribution and prioritization of species and areas for conservation, but evolutionary history is rarely considered in practical conservation activities. One impediment to implementation is that arguments related to the human‐centric benefits of evolutionary history are often vague and the underlying mechanisms poorly explored. Herein we identify the arguments linking the prioritization of evolutionary history with benefits to people, and for each we explicate the purported mechanism, and evaluate its theoretical and empirical support. We find that, even after 25 years of academic research, the strength of evidence linking evolutionary history to human benefits is still fragile.

Most – but not all – arguments rely on the assumption that evolutionary history is a useful surrogate for phenotypic diversity. This surrogacy relationship in turn underlies additional arguments, particularly that, by capturing more phenotypic diversity, evolutionary history will preserve greater ecosystem functioning, capture more of the natural variety that humans prefer, and allow the maintenance of future benefits to humans. A surrogate relationship between evolutionary history and phenotypic diversity appears reasonable given theoretical and empirical results, but the strength of this relationship varies greatly. To the extent that evolutionary history captures unmeasured phenotypic diversity, maximizing the representation of evolutionary history should capture variation in species characteristics that are otherwise unknown, supporting some of the existing arguments. However, there is great variation in the strength and availability of evidence for benefits associated with protecting phenotypic diversity. There are many studies finding positive biodiversity–ecosystem functioning relationships, but little work exists on the maintenance of future benefits or the degree to which humans prefer sets of species with high phenotypic diversity or evolutionary history. Although several arguments link the protection of evolutionary history directly with the reduction of extinction rates, and with the production of relatively greater future biodiversity *via* increased adaptation or diversification, there are few direct tests. Several of these putative benefits have mismatches between the relevant spatial scales for conservation actions and the spatial scales at which benefits to humans are realized. It will be important for future work to fill in some of these gaps through direct tests of the arguments we define here.

## I. INTRODUCTION

*To a conservationist, regardless of relative abundance, is Welwitschia equal to a species of Taraxacum? Is the panda equivalent to one species of rat?* Atkinson (1989) *answered this question in the following way: ‘given two threatened taxa, one a species not closely related to other living species and the other a subspecies of an otherwise widespread and common species, it seems reasonable to give priority to the taxonomically distinct form’*. (Vane‐Wright, Humphries & Williams, 1991)

Given the anthropogenic threats of habitat loss, fragmentation, biological invasions, pollution, and climate change, the call for effective and efficient conservation has never been stronger (Ceballos & Ehrlich, 2002; Barnosky *et al*., 2011; Pimm *et al*., 2014; Díaz *et al*., 2015). The grave nature of these threats and their global extent must be reconciled with the limited resources available for conservation. Scientific research plays an important role in identifying and verifying explicit goals for systematic conservation plans (Margules & Pressey, 2000). However, our understanding of the full impacts of humanity on, and the benefits for humanity from, biodiversity is still incomplete (e.g. Chapin *et al*., 2000; Luck, 2007; Heller & Zavaleta, 2009; Wardle *et al*., 2011; Cardinale *et al*., 2012). In addition, the links between biodiversity and the processes occurring in natural ecosystems are incompletely documented (McNeely *et al*., 1990; Purvis & Hector, 2000; Mace, Norris & Fitter, 2012). This greatly complicates the task of selecting among various targets (e.g. species, sites) or prioritizing different facets of biodiversity (e.g. genetic diversity, species richness, or phenotypic diversity). Proposed solutions to this ‘agony of choice’ (Vane‐Wright *et al*., 1991) must address the concurrent limitations of funding, feasibility, and knowledge regarding the relative values of individual targets.

One solution might be to consider measures of evolutionary history (see Table 1 for glossary) to identify sites or taxa with particular conservation value. This evolutionary‐history‐based approach was first formalized in the 1990s (Vane‐Wright *et al*., 1991 *;* Crozier, 1992, 1997 *;* Faith, 1992 *;* Williams *et al*., 1994 *;* Weitzman, 1998). The message of these early papers was that sets of species with more evolutionary history represent greater biodiversity, and so should receive higher conservation priority. More recent expressions of the idea include reference to the ‘phylogenetic gambit’ (Mazel *et al*., 2018), which proposes that evolutionary history could be (but is not always) an efficient approach to capturing a wide variety of form and function without needing to quantify the multitude of species' traits, ecological strategies, and contributions to ecosystem function.

**Table 1:** Glossary of terms.

*Complementarity*	(*i*) The increase in performance or function of a group of species as compared to their performances in monoculture, resulting from niche partitioning (ecological context). (*ii*) The principle of iteratively selecting new units (e.g. sites, species) for prioritization to maximize the inclusion of new attributes (conservation context).
*Ecosystem function*	Any biological, chemical, and physical processes that are components of an ecosystem, where ecosystem is broadly defined as a biological community of interacting organisms and their physical environment.
*Ecosystem benefit*	Direct or indirect benefit that people obtain from the functioning of ecosystems and/or the existence of particular biological entities. Corresponds to ecosystem services according to the Millennium Ecosystem Assessment or to many of nature's positive contributions to people according to the IPBES (Díaz *et al*., 2018).
*Evolutionary distinctiveness*	The amount of non‐redundant evolutionary change associated with a given taxon. Taxa with fewer close relatives and on longer branches of a phylogeny are scored as being more evolutionarily distinctive.
*Evolutionary history*	The total amount of evolutionary change represented by a set of taxa. Usually quantified using metrics of phylogenetic diversity (PD).
*Evolutionary potential*	Potential of a set of taxa for future evolutionary change or diversification.
*Human‐centric value*	In this review, the purported value of biodiversity that is dependent (directly or indirectly) on human consideration. Human‐centric value captures both instrumental and relational values, as considered by the IPBES.
*Intrinsic value*	Purported value of biodiversity that is independent from human consideration. Sometimes synonymous with ‘inherent value’.
*Option value*	A hypothesised measure of the degree to which the maintenance of future benefits to people is contingent on contemporary biodiversity. Generally, the nature of the future benefits is undefined and the timing of the need for their delivery is uncertain.
*Phenotypic diversity*	The total range of phenotypic variation in a set of taxa, which can include molecular, physiological, phenological, behavioural or morphological characteristics. We focus on measures of the range of trait values or trait states in a set [generally measured as trait richness (TR)], rather than measures that capture trait evenness or divergence.
*Phylogenetic diversity*	A measure of the total evolutionary history represented by a set of taxa. Calculated by summing the branch lengths connecting a set of taxa on a phylogeny. These branch lengths can represent elapsed time or some measure of genetic or phenotypic change.
*Phylogenetic signal*	The ability of shared phylogenetic relationships to predict the covariance of a trait across of a set of species. Generally considered as being due to related species inheriting traits from a common ancestor.
*Portfolio/Insurance effect*	Reduction of spatial or temporal variability in the functioning or performance of a system through greater diversity. This assumes that having a larger portfolio of traits maximizes the differential responses to changing conditions.
*Sampling effect*	The statistical effect by which the probability that an object with the characteristic of interest (e.g. a species with a particular trait) is present increases as the variety of objects sampled increases.

Modern conservation biology does consider multiple facets of biodiversity (Purvis & Hector, 2000; Mace, Gittleman & Purvis, 2003); the concept of evolutionary history is undeniably influential, underpinning a wide variety of scholarly books (Purvis, Gittleman & Brooks, 2005; Pellens & Grandcolas, 2016), analyses, and recommendations about the distribution and prioritization of species and areas for conservation based on their representation of the tree of life (e.g. Forest *et al*., 2007; Isaac *et al*., 2007; Tucker *et al*., 2012; Jetz *et al*., 2014; Pollock, Thuiller & Jetz, 2017; Cadotte & Tucker, 2018). Despite the potential value of evolutionary history, most of this work has remained within the academic literature and is rarely applied to existing or new conservation activities. One notable exception is the EDGE of Existence program at the Zoological Society of London, which uses a compound measure of evolutionary distinctiveness (so, highlighting species without close relatives) and conservation status to develop lists identifying priority species (Isaac *et al*., 2007). Typically though, the metrics, proposed reserve networks, and resulting maps produced in this literature are still waiting to be applied to conservation planning and funding activities.

This gap between concept and application has been noted (notably by Winter, Devictor & Schweiger, 2013), but remains to be bridged. One past limitation was the large and often confusing collection of indices of evolutionary history, since these can lead to a variety of different answers to the same question. Recent work has clarified the interpretation of, and relationships between, metrics (Pavoine & Bonsall, 2011; Tucker *et al*., 2017), making the choice of measure clearer. Even so, the benefits to people associated with protecting evolutionary history (especially as compared to some alternative prioritization approach) have not been demonstrated sufficiently, and the arguments are generally logical (see, e.g. Lean & Maclaurin, 2016) rather than based on direct evaluation of evidence. In this review, we focus exclusively on the human‐centric benefits of evolutionary history, as these can be measured and evaluated using currencies of interest for conservation [i.e. the number of valued biological attributes or characters (Williams, Gaston & Humphries, 1994)]. There are important philosophical discussions about the intrinsic (or inherent) value of biodiversity that can be found in the conservation biology and philosophy literatures (see, e.g. Agar, 2001; Norton, 2003; Maclaurin & Sterelny, 2008; Maier, 2012; Faith, 2017; Newman, Varner & Linquist, 2017). However, these values are in essence unquantifiable.

For evolutionary history to be a useful solution to the agony of choice faced by conservation practitioners, it must receive a comprehensive assessment to clarify when and how it benefits people. To that end, we ask a specific question: does protecting evolutionary history provide quantifiable benefits for people? We (*i*) develop a framework identifying and generalizing the existing arguments for the conservation benefits of evolutionary history, (*ii*) assess the strength of theoretical and empirical support for these arguments, and, importantly, (*iii*) outline avenues for directed research to address gaps in existing knowledge. We hope that this framework will inspire more focussed research in the field and ultimately aid conservation practitioners in evaluating the benefits of an evolutionarily informed approach.

## II. A FRAMEWORK IDENTIFYING THE CONSERVATION VALUE OF EVOLUTIONARY HISTORY

**Figure 1:**
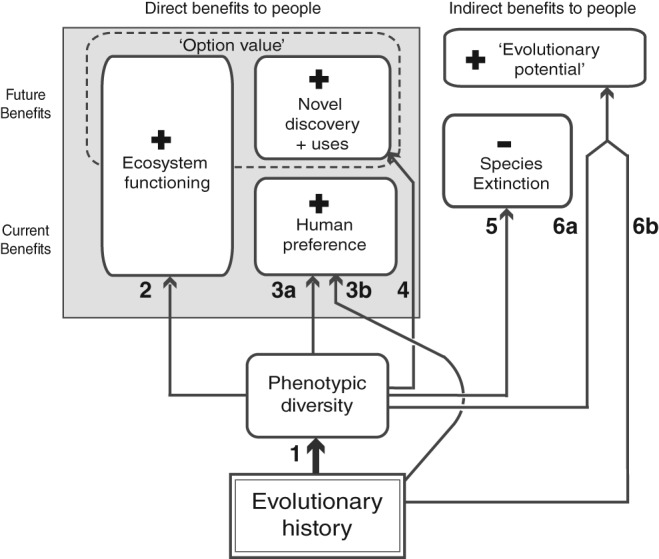
Conceptual figure showing the proposed relationships between evolutionary history and six potential benefits to people (rounded boxes). Links between evolutionary history and conservation outcomes are shown with labelled arrows 1–6. Note that the majority of relationships with evolutionary history are indirect and mediated *via* a single direct potential linkage between evolutionary history and phenotypic diversity (Link 1). Each link represents a relationship that is a testable hypothesis: when two hypotheses are possible, one mediated by phenotypic diversity and one directly related to evolutionary history, these are identified as *a* and *b*. The temporal scales at which benefits occur are indicated, moving from current benefits to those that may be achieved at some future time.

We identified six general arguments for conservation actions focused on preserving greater evolutionary history. More evolutionary history has been said (1) to capture more phenotypic diversity present in a set of species – this is the foundational assumption; (2) to lead to enhanced benefits from ecosystem processes; (3) to enhance human experience due to a preference for nature's variety or novelty; (4) to maintain potential future uses for biodiversity; (5) to lead to decreased extinction rates; and (6) to lead to increased adaptability and future biodiversity production (evolutionary potential). We do not presuppose the relative importance of any argument. Each of the arguments (Links 1–6) is represented in Fig. 1 as a connecting arrow between putative benefits (rounded boxes). We discuss the relevant mechanisms by which these benefits could arise, the scales at which they might be relevant, and evaluate the empirical and theoretical support in the literature. Arguments are summarized in Table 2.

**Table 2:** Summary table reflecting the logic, support for or against, and future directions for each argument regarding the human‐centric benefits of evolutionary history

	**Link 1**	**Link 2**	**Link 3**	**Link 4**	**Link 5**	**Link 6**
	***Does evolutionary history have surrogacy value for phenotypic diversity?***	***Does phenotypic diversity contribute to the maintenance of ecosystem functioning for both the present and future?***	***Do humans have preferences for evolutionary history and/or phenotypic diversity?***	***Can evolutionary history and phenotypic diversity provide future options for humanity?***	***Does greater evolutionary history or phenotypic diversity reduce species' extinction risk?***	***Does evolutionary history and phenotypic diversity provide greater evolutionary potential?***
**Relevant concepts**	Models of trait evolution, phylogenetic signal	Biodiversity—ecosystem functioning, sampling effect, complementarity	Biophilia, socio‐cultural, spiritual, philosophical, or historical factors	Option value, future value of biodiversity	Sampling effect, traits associated with extinction risk	Potential for future diversification or adaptation, speciation rate
**Key points**	Simple models of trait evolution predict that PD should act as a surrogate for TR, and the surrogacy increases with more traits. Surrogacy is unknown for more complex models. Phylogenetic signal is common but such signal is insufficient to predict the full strength of the surrogacy of PD for TR. Surrogacy value of PD for TR at one spatial scale (global, regional) may not reflect the surrogacy observed in a subset of those taxa (e.g at local scale) due to subsets being non‐ random.	The relationship between evolutionary history and ecosystem functioning is dependent on the exact mechanism by which phenotypic differences among species translate into differences in functioning. The relationship between biodiversity—including TR—and ecosystem functioning is typically positive, although it is not necessarily strong and rarely linear. The scale at which phenotypic diversity is protected is not necessarily the only scale at which it will affect functioning.	Further research on human preference for PD or TR is necessary. Studies must differentiate between human preference for PD or TR, *per se*, and taxonomic biases (i.e. for top predators, megafauna, and mammals), sampling effects, preferences for rarity, etc. Human preference is a critical driver of conservation action, and so this is an important link to study, both phenomenologically and with regard to mechanism.	Future impacts of biodiversity may include potential threats as well as benefits. The current benefits *versus* costs of TR (Links 2 and 3 in Fig. 1) may provide a useful basis for estimating the future distribution of costs/benefits. Under changing conditions, sets of species with greater PD or TR may—*via* a sampling effect—be more likely to include members suited to the new environment and so provide resilience against loss of human benefits. True tests are difficult given the long time scales necessary, although work in microcosms and species with rapid generation times could be insightful.	Preserving greater phenotypic diversity can preserve key traits associated with extinction risk. Further research is necessary to understand the balance between preserving species with traits that increase *versus* decrease extinction risk. On ecological time scales, greater PD or TR can reduce extinction by stabilizing systems through spatial and temporal insurance effects. The relationship between evolutionary history, phenotypic diversity, and extinction risk differs among groups of species. Identifying general predictors of this relationship is key.	Species that contribute disproportionately to PD might also be species with high genetic variation and thus greater adaptive potential. Alternatively, current evidence is consistent with the notion that high‐PD sets of species capture ‘dead clades walking’. Existing evidence is insufficient to link evolutionary history or phenotypic diversity to evolutionary potential measured as adaptation or diversification.
**Further questions**	How do macroevolutionary and biogeographic processes interact with ecological assembly to determine the relationship between PD and TR at smaller spatial scales? How strong is the phylogenetic signal in traits specifically of conservation interest and which models of trait evolution most frequently generate these traits?	Does PD act as a surrogate for traits specifically known to drive ecosystem functions? Are positive relationships between TR and ecosystem functioning similar for traits, taxa, and systems beyond those typically studied? E.g. vertebrates, higher trophic levels, larger spatial scales.	Do people favour protected areas harbouring sets of distantly related species more than protected areas that are home to sets of closely related species? Do people specifically prefer species with no close relatives and, if so, why?	Find examples of biodiversity conservation that can be used to evaluate the option value associated with different regimes of biodiversity protection. Is there relevant research on option value as considered here in other disciplines?	Is there a general mechanism relating phenotypic diversity and extinction risk, across clades and ecosystems? Do evolutionarily distinct species tend to be at higher, or lower, risk of extinction?	Do sets of taxa chosen to maximize PD differ systematically in their genetic diversity and ability to adapt and diversify? Does the fossil record contain signals linking standing PD and subsequent diversity?

PD, phylogenetic diversity; TR, trait richness.

## III. MEASURING EVOLUTIONARY HISTORY

**Figure 2:**
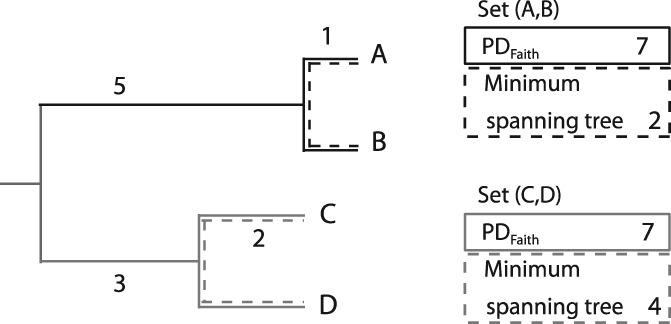
Comparing measures of phylogenetic diversity (PD). Faith's phylogenetic diversity (PD_Faith_) is the sum of the branch lengths on the minimum spanning tree linking a set of terminal taxa to the root. This inclusion of an unnamed root taxon results in an implicit complementarity aspect to PD_Faith_. Not all definitions of PD include the root, which avoids this complementarity issue, but has created a source of confusion in the literature. Values indicate branch lengths – note here that the tips are all not the same distance from the root; species are identified with letters shown at tips. PD_Faith_ for sets (A,B) and (C,D) would be 7. A PD measure that sums just the branch lengths on the minimum spanning tree would lead to a value of 1 + 1 = 2 for the (A,B) subset and 2 + 2 = 4 for the (C,D) subset.

There are many measures available to quantify evolutionary history, which can be used for the prioritization of both individual taxa and sets of taxa (for a comprehensive review, see Tucker *et al*., 2017). We focus on measures calculated using a phylogeny – a branching diagram describing hierarchical descent with modification amongst a group of taxa. The majority of measures used in the conservation literature consider the sum of the branch lengths along a phylogeny connecting a set of taxa [Crozier, 1992; Faith, 1992; see Vane‐Wright *et al*. (1991) for a version that does not incorporate branch lengths]: these are referred to as indices of phylogenetic diversity (PD). Note that in many situations, PD is strongly correlated with the number of species in the set of taxa. Some versions of PD do not include the root of the tree, in which case only the sum of the branch lengths linking the terminal taxa is used (dashed lines, Fig. 2) (Crozier, 1992); when a set of taxa spans the root of the tree, the measures are equivalent. Faith's PD (Faith, 1992) is the most common and lasting version of PD: it sums the branches linking a set of terminal taxa to the root (Fig. 2).

Sets containing more distantly related taxa (i.e. taxa separated by longer branches on a phylogeny) capture more evolutionary history and so have larger PD values than sets of closely related taxa. The units of the branch lengths determine the precise interpretation of PD – each branch may have length set to 1, or branch lengths may be in units of observable changes in traits (or ‘features’; Faith, 1992), or genetic distances based on the markers used to infer the phylogeny, or inferred time, as estimated with clock models tied to fossil calibrations. However, PD indices are frequently described more generally (regardless of phylogeny type) as estimates of total ‘evolutionary history’, ‘evolutionary information’, or ‘divergence’. All these types of branch‐length measures have been used in conservation analyses.

PD is certainly not the only measure of evolutionary history to be applied in conservation. Other relatively common measures include indices that weight branches by some measure of the abundance of descendent species, such as phylogenetic endemism (Rosauer *et al*., 2009), indices of species‐specific evolutionary distinctiveness (ED), indices that combine distinctiveness with weighting for species threat (Redding & Mooers, 2006; Isaac *et al*., 2007), and various indices that incorporate within‐tip genetic diversity (Carvalho *et al*., 2017). Our focus on PD reflects its place as the foundational and most commonly applied measure in the literature. It is also conceptually simple, representing the total diversity captured by the phylogeny. Regardless, many of the arguments presented here for the value of PD will be relevant to other metrics of evolutionary history.

## IV. BENCHMARKING THE PERFORMANCE OF INDICES OF EVOLUTIONARY HISTORY

Supporting the arguments in Fig. 1 requires that there is a mechanism or empirical evidence associated with that linkage, and also that evolutionary history performs better in achieving this human‐centric benefit relative to some alternative approach for identifying conservation targets. Choosing such a benchmark is not simple. One approach common in the literature – and the one we use here – is to compare the total response achieved by selecting *n* species to maximize PD with that achieved using a random set of *n* species (e.g. Rodrigues, Brooks & Gaston, 2005). This is not because real conservation decisions conserve species or areas randomly, but rather that alternative prioritization schemes are expected to be unbiased with respect to evolutionary history. Where a response is measured for multiple sets or areas, the average performance (in terms of the response value of interest) of PD can be compared to the average achieved using many random selections, and the variation around that average. One limitation of any benchmark based on the average performance is that if the maximum PD set is compared to multiple replicates (e.g. multiple random samples of *n*), there may be meaningful variation such that any single comparison could perform much better or much worse. To complicate matters further, there are usually multiple sets of *n* species that maximize PD.

Other benchmarks are possible. For example, several papers have asked whether selecting sets of species that maximize PD also maximizes other currencies of interest (i.e. have the highest phenotypic diversity, or the lowest extinction rates). For example, Pollock *et al*. (2017) explored whether prioritization of sites to maximize different facets of diversity led to congruent protection of global hotspots of evolutionary history, phenotypic diversity, and/or species richness. Although identifying hotspots is a highly relevant conservation goal, it is unknown whether the congruence of extremes, such as hotspots, is more or less likely than considering average expectations as we do here (but see e.g. Grenyer *et al*., 2006).

## V. EVALUATING THE ARGUMENTS FOR EVOLUTIONARY HISTORY

### (1) Does evolutionary history have surrogacy value for phenotypic diversity?

#### (a) *Rationale*

The most commonly expressed argument for the utility of evolutionary history is that it is a useful – although probably imperfect – surrogate for the phenotypic diversity represented by a set of species. This line of reasoning is implicit in many of the earliest arguments, which touted the protection of evolutionary history precisely for its ability to capture as wide a variety of form and function as possible (Faith, 1992). This assumes that as the total PD captured by a set of species increases, the expected amount of phenotypic divergence between them should increase as well. Link 1 states that choosing species that maximize evolutionary history (e.g. measured using PD) will encompass more phenotypic diversity compared to a randomly selected set of species. Table 3 summarizes the evidence for this argument.

**Table 3:** Theoretical predictions and empirical evidence for the surrogacy of evolutionary history [*via* phylogenetic diversity (PD)] for phenotypic diversity [*via* trait richness (TR)]

**(1) What are the expectations?**	**Relevant questions**	**Example test**	**General findings**	**References**
Traits have phylogenetic signal	Do traits typically have phylogenetic signal?	Using observed data: across a wide range of traits and species, Blomberg *et al*. (2003) found that 92% of traits exhibited significant phylogenetic signal.	The presence of a phylogenetic signal in traits is ubiquitous, but variable in strength. Particular traits can have a weak or negligible signal. It is not known whether classes of traits (e.g. those associated with ecosytem function or known use) show more or less signal than average.	Blomberg *et al*. (2003); Zheng *et al*. (2009); and many others.
Models of trait evolution predict correlations between PD and TR	Do models of trait evolution result in predictable and strong relationships between evolutionary history and phenotypic diversity?	With simulations: for common models (BM, OU), Tucker *et al*. (2018) found that both models produce correlations between PD and TR, and that these correlations are stronger when additional traits are included in TR.	Simple models of trait evolution generate correlations between PD and TR. As models increase in complexity the correlation will be more variable and often weaker. A phylogenetic signal does not necessarily predict good surrogacy.	Tucker *et al*. (2018); Mazel *et al*. (2017); Kraft *et al*. (2007); O'Meara (2012).
	Do models of trait evolution typically fit observed trait data and phylogenetic trees?	Using observed data: Pennell *et al*. (2015) found that an OU model was highly supported (compared to other common models) over 337 data sets, but all the models typically used were often inadequate.	Models that include phylogeny typically outperform those that do not. It is not known whether the commonly used models are sufficient to describe trait evolution and make predictions about PD and TR.	Pennell & Harmon (2013); Harmon *et al*. (2010); Pennell *et al*. (2015).

**(2) What do empirical comparisons show?**				
Observed relationships between PD and TR	Across multiple sites, does site PD correlate with site TR?	Using observed data: Dehling *et al*. (2014) found that trends in PD change and TR change with elevation were congruent for frugivorous birds in South America, but species richness was not controlled for.	The relationship between observed site PD and TR is generally positive, and often strong, however it varies significantly among systems, and is generally mediated by species richness.	Devictor *et al*. (2010); Dehling *et al*. (2014); Tribot *et al*. (2016); Albouy *et al*. (2017); Remeš & Harmáčková (2018); many others.
	Do species chosen to maximize PD, have greater TR than species chosen at random?	Using observed data: Mazel *et al*. (2018) showed that at the global and regional scale, sets of species chosen to maximize the protection of PD lead to better‐than‐random protection of TR, but with great variation around the mean.	Maximizing PD should protect greater‐than‐average TR, but exceptions occur, e.g. when there is strong niche conservatism or rapid niche differentiation.	Forest *et al*. (2007); Mazel *et al*. (2018).
	Do sites chosen to maximize PD have greater TR than sites chosen at random?	No known test.	NA	NA
	Do sites chosen to maximize PD also maximize TR?	Using observed data: Albouy *et al*. (2017) showed that hotspots of PD and TR have low congruency.	The literature rarely finds congruence between areas with maximal PD and maximal TR.	Albouy *et al*. (2017); Brum *et al*. (2017); Safi *et al*. (2011); Pollock *et al*. (2017); Mazel *et al*. (2014).
	Do species chosen to maximize PD also maximize TR?	Using observed data: Kelly, Grenyer & Scotland (2014) showed that the species separated by the most distance on a tree often do not show maximal trait divergence.	Few studies directly test this prediction.	Kelly *et al*. (2014).
	Do smaller sets of species have more variable correlations between PD and TR?	Using observed data: Mazel *et al*. (2018) looked at the surrogacy of PD for TR and found that surrogacy is most variable when small numbers of species are chosen.	Few studies directly test this prediction.	Pollock *et al*. (2017); Mazel *et al*. (2018).
The correlation between PD and TR depends on set properties.	Do biologically relevant samples disrupt the correlation between PD and TR?	Using observed data: Prinzing *et al*. (2008) showed that communities with more plant lineages had less phenotypic diversity than communities with fewer plant lineages as a result of non‐random assembly processes.	Few studies directly test this prediction. Ecological processes are typically expected to lead to non‐random sets of species, but this may only sometimes lead to altered relationships between TR and PD.	Prinzing *et al*. (2008); Cavender‐Bares, Keen & Miles (2006).

BM, Brownian motion; NA, not applicable; OU, Ornstein‐Uhlenbeck.

Identifying and describing the mechanisms behind this relationship between phenotypic diversity and measures of evolutionary history is important, but has been hampered by differing definitions and assumptions. In its original form (Faith, 1992), PD was explicitly said to capture ‘feature diversity’, where ‘features’ are restricted to the variety of states across homologous characters. However this is a specific and perhaps even circular model for how PD and trait states relate (see Faith, 1992, 2016). Alternatively, in the ecological literature, PD is more often expected to relate to phenotypic diversity defined as the total range of values present in a set of taxa for any measurable trait (McGill *et al*., 2006; Violle *et al*., 2007), including physiological, phenological, morphological, and behavioural traits. This definition of traits includes, but is not limited to those defined as ‘functional’, i.e. those associated with individual performance (see discussion in Mazel *et al*., 2019; Owen *et al*., 2019). This conceptualization of phenotypic diversity can be measured using trait richness (TR) indices (Pavoine & Bonsall, 2011), i.e. the total variation in single or multiple traits in a set of species (Cornwell, Schwilk & Ackerly, 2006; Villéger, Mason & Mouillot, 2008; Blonder *et al*., 2014). Importantly, ‘feature diversity’ measured as the sum of the unique character states present in a set of species can be represented as TR for a single discrete trait. For the remainder of this review, unless otherwise noted, we use the term ‘trait’ to capture the more general concept of phenotype.

The expected relationship between phenotypic diversity (measured using TR) and total evolutionary history (measured using PD) can be described using macroevolutionary models of evolution (O'Meara, 2012; Pennell & Harmon, 2013; Tucker *et al*., 2018). This provides insight into the drivers of empirical relationships between PD and TR and allows prediction about species' differences that are not yet measured. We focus on this definition of the relationship between phenotypic diversity and evolutionary history (and between PD and TR), as it best aligns with the existing literature on traits and their many purported benefits. Note, while other dimensions of phenotypic diversity [such as trait evenness or trait divergence (Villéger *et al*., 2008)] may also be relevant to ecosystem processes or to other benefits, these do not align directly with PD (which is a richness measure (Pavoine & Bonsall, 2011; Tucker *et al*., 2017), and so these alternative measures are not considered here.

Theoretical models of trait evolution that include phylogeny provide some general expectations for how TR might change with PD, and these can be fitted to empirical data to evaluate which models might be more likely. Outputs from these models provide significantly better fits to observed trait variation than those which do not consider phylogeny, with unbounded Brownian Motion and the Ornstein–Uhlenbeck models most often identified as the best descriptors of existing data (Harmon *et al*., 2010; Pennell *et al*., 2015). This suggests that PD has the potential to be a good surrogate for TR. However, even if TR is tied to evolutionary history, the true relationship might reflect far more complex dynamics than simplistic models: the best‐fitting model may still not be a particularly good description of trait change (Pennell *et al*., 2015), and several (even mutually contradictory) models may appear to fit a phylogeny and a set of trait values at the tips equally well (Revell, Harmon & Collar, 2008). When measures of phenotypic diversity incorporate multiple traits, there is evidence that – at least for common models of trait evolution – PD should become a better surrogate for TR as more traits are incorporated (Tucker *et al*., 2018). The latter authors also found that for more complex models of trait evolution, including those with multiple rates among clades or through time, or multiple optima, this surrogacy weakens (Tucker *et al*., 2018). Typically, simple models of trait evolution predict that PD should act as a surrogate for TR, but this is not universally true for all models of trait evolution.

One factor in understanding the relationship between PD and TR, and the performance of PD as a surrogate, is the strength of the phylogenetic signal for traits of interest. The performance of PD as a surrogate should improve as the phylogenetic signal increases, with the phylogenetic signal for a trait varying from non‐existent to very strong depending on the generating model of evolution and, importantly, on the measure of phylogenetic signal used (Münkemüller *et al*., 2012). Studies of observed traits generally find a phylogenetic signal, but the strength varies greatly depending on the type of system and trait considered (Blomberg, Garland & Ives, 2003; Zheng *et al*., 2009). Phylogenetic signal can also be measured across multiple traits, which may be especially relevant for predicting surrogacy for multi‐trait measures of TR (Klingenberg & Gidaszewski, 2010; Adams, 2014): this multi‐trait signal is likely to vary depending on the underlying models and the measure used, and on whether component traits evolve independently or in a correlated fashion. The observation that a phylogenetic signal is common highlights that a surrogacy relationship between PD and TR is possible at least for some systems and traits, but is insufficient to predict the strength of that relationship.

Even if there is a strong phylogenetic signal for traits of interest across the entire phylogeny, non‐random subsets of the tree can have variable strengths of phylogenetic signal, weakening the link between such a signal and surrogacy. For example, even with a phylogenetic signal, it is possible for a subset of species that maximizes PD to capture less TR than a random set of species of the same size (Mazel *et al*., 2017). Mazel *et al*. (2017) showed that this is because the subset of species that maximizes PD tends to occupy a non‐random position in phylogenetic and trait space. For example, when selected from a species pool with an imbalanced phylogeny and a speciational model of evolution, the species that maximize PD are phylogenetically distant but phenotypically similar. In addition, non‐random subsets of species in a site or region are likely the rule, not the exception, since ecological assembly processes select for non‐random combinations of species with respect to their phylogenetic relationships and/or traits (Webb *et al*., 2002; Cavender‐Bares *et al*., 2009; Gerhold *et al*., 2015; Cadotte, Davies & Peres‐Neto, 2017). Also, if subsets of species are relatively small, stochasticity in composition could lead to significant variation in the surrogacy value of PD. It is important to recognize that even when the surrogacy value of PD for TR is observed at regional or global scales, this relationship may not hold for subsets that reflect sorting in specific habitats.

The choice of benchmark to compare PD to, and the metric of TR, can both lead studies to divergent conclusions. We strongly recommend the use of metrics that measure total (summed) diversity [PD and various measures of TR], since this allows direct comparison between diversity facets (see e.g. Villéger *et al*., 2008; Pavoine & Bonsall, 2011; Blonder *et al*., 2014). Using the random benchmark described previously, Mazel *et al*. (2018) compared PD and TR across a small number of ecologically relevant traits for bird, mammal, and tropical reef fish species. They found that PD outperformed a random selection of species, capturing 18% more TR, although the relationship was weakened when clades showed recent trait divergence or strong trait conservatism. Despite this successful performance on average, there were wide confidence intervals on the surrogacy measures and any individual subset of species that maximized PD could perform much worse or better as a surrogate than random expectation. Other studies have chosen different benchmarks (e.g. Devictor *et al*., 2010; Pollock *et al*., 2017), and these tend to provide more varied conclusions about the surrogacy value of PD. Studies that consider the congruence of hotspots of evolutionary history *versus* phenotypic diversity, or that attempt to maximize the protection of both TR and PD in existing protected areas, generally find important mismatches between areas of maximum PD and areas of maximum TR, at large geographic scales and for a variety of taxa (Devictor *et al*., 2010; Safi *et al*., 2011; Spasojevic & Suding, 2012; Dehling *et al*., 2014; Mazel *et al*., 2014; Albouy *et al*., 2017; Brum *et al*., 2017; Pollock *et al*., 2017). The choice of metric of diversity, number of traits, and benchmark greatly affects support for surrogacy arguments.

#### (b) *Conclusions and future directions*

Given the complex connections between the outcomes of models of trait evolution, non‐independence of many traits, and the types of traits of particular conservation interest, no single general relationship between PD and TR is expected. We conclude that if evolutionary history is to be used as surrogate of phenotypic diversity, the definition of the two terms must be such that each is measurable and quantitative. Of course, as data on TR accumulate, the need for a surrogacy measure may diminish, and so much also hinges on the ability to identify and measure traits that contribute to the links in Fig. 1 directly. There is good evidence to support the general conclusion that PD is a more effective surrogate for TR – on average – than a phylogenetically uninformed choice of species (Mazel *et al*., 2017; Tucker *et al*., 2018). However, the strength of this surrogacy is quite variable when evaluated empirically, and the variance is often high (Mazel *et al*., 2018). An important goal for future work is to identify the distribution describing the surrogacy of PD for TR (Mazel *et al*., 2019), and potential predictors of weaker or stronger surrogacy relationships, including the number of traits, and their correlations. Incorporating potential uncertainty more generally into, e.g. spatial prioritization activities would allow plans to include confidence intervals on the estimated surrogacy of a set of PD‐maximal species for TR. Other important questions relate to the intersection of ecological and evolutionary processes with each other and with conservation: how strong is the phylogenetic signal in traits that are of conservation interest (e.g. beyond body mass in vertebrates)? And which models of trait evolution most frequently generate these trait distributions? And, for Links 2, 3, 5, and 6 (see Sections V.2, V.3, V.5, and V.6), how do macroevolutionary and biogeographic processes interact with ecological assembly to determine the relationship between PD and TR at smaller spatial scales? Such research will improve our understanding of the expectations for PD‐TR surrogacy, and better inform arguments about the conservation value of evolutionary history in general.

### (2) Do evolutionary history and phenotypic diversity ensure the maintenance of ecosystem functioning for both the present and future?

#### (a) *Rationale*

If phenotypic diversity among species reflects differences in ecological strategies among species (Violle & Jiang, 2009), measures of total TR should be a useful surrogate for the potential contribution of those species to ecosystem functioning. Link 2 states that a set of species with higher phenotypic diversity (measured *via* TR) should better maintain ecosystem functioning (EF): declines in TR could lead to the loss of phenotypic diversity relevant to ecosystem processes such as biomass production, nutrient cycling, decomposition, productivity, or resilience in the face of disturbance (Walker, Kinzig & Langridge, 1999; Solan *et al*., 2004; Petchey & Gaston, 2006; Reich *et al*., 2012). Evaluating this potential relationship between phenotypic diversity and ecosystem functioning requires consideration of the relevant spatial scale for analysis, the processes of interest, and the structure of the trait–function relationships.

Positive biodiversity–ecosystem function relationships are thought to arise because greater species richness leads to the accumulation of greater variation in form and function, which can be estimated using indices of phenotypic diversity such as TR (Díaz & Cabido, 2001; Loreau *et al*., 2001). Species' traits, and the total range of traits in an assemblage can contribute to ecosystem functions *via* two main mechanisms: sampling and complementarity effects. The first (also known as a selection effect) refers to the fact that a group of species with more TR is more likely to include species with traits which could have direct impacts on functioning or drive competitive outcomes (Tilman, Wedin & Knops, 1996; Loreau, 1998). For example, sets with greater TR are, by chance alone, more likely to include traits that directly impact function (Lavorel & Garnier, 2002; Díaz *et al*., 2007; Lavorel *et al*., 2011; Cadotte, 2017), like a nitrogen‐fixing plant that improves soil nitrogen availability (e.g. Hector *et al*., 1999; Davies *et al*., 2016) or a specialized pollinator or disperser that improves fruit production (Galetti *et al*., 2001; Vanbergen, 2013). Alternatively, the complementarity argument states that greater TR might result in greater ecosystem function directly through complementary use of available resources (Díaz & Cabido, 2001; Díaz *et al*., 2007; Cadotte, 2017), which results in a more thorough conversion of the total resource pool into measurable functioning (Loreau, 1998; Loreau & Hector, 2001; Fargione & Tilman, 2005; Hooper *et al*., 2005; Turnbull *et al*., 2016).

Note that even if Link 1 is true and there is a strong relationship between evolutionary history and phenotypic diversity, evolutionary history could poorly predict actual ecosystem functioning if the mechanisms that relate phenotypic diversity (measured by TR) to ecosystem functioning are only weakly captured by the phylogeny. For example, PD is only weakly related to average pairwise differences among species (Tucker *et al*., 2017) and so may poorly predict ecosystem function if such function is governed by independent pairwise interactions. Alternatively, PD might be a better direct predictor of ecosystem functioning than measured TR if many traits that affect complementarity are unknown, but the sets of species with high PD show higher set‐wise complementarity or lower average competition. The relationship between evolutionary history and ecosystem functioning is dependent on the exact mechanism by which phenotypic differences among species translate into differences in such functioning.

Much research effort has already been dedicated to evaluating the relationship between biodiversity and ecosystem functioning (Loreau *et al*., 2001; Hooper *et al*., 2005; Balvanera *et al*., 2006; Cardinale *et al*., 2006, 2012), although studies disproportionately consider species richness rather than traits, a small number of functions (e.g. biomass), and highly simplified species assemblages (Wardle, 2016). Empirical studies do often find positive relationships between measures of biodiversity and ecosystem functioning. However, results vary among systems and processes, and weak, absent or even negative relationships have also been reported (Wardle, Bonner & Nicholson, 1997; Duarte, 2000; Balvanera *et al*., 2006; Cardinale *et al*., 2006, 2007, 2012; Tilman, Isbell & Cowles, 2014). Phenotypic diversity is hypothesized to underlie many of the positive biodiversity–ecosystem functioning relationships (Tilman *et al*., 1997; Hodgson *et al*., 1998; Tilman, 1999; Suding *et al*., 2005; Mokany, Ash & Roxburgh, 2008), and indeed, the range of trait values (TR) present in a system is a good predictor of ecosystem functioning in several studies (Tilman *et al*., 1997; Petchey & Gaston, 2006; Naeem, Duffy & Zavaleta, 2012). Interestingly, Cadotte *et al*. (2009) found that PD was a better direct predictor of functioning than both trait and species richness measures, and they proposed that this was due to the potential that ecological differentiation scaled with PD better than did trait‐based measures (see also Flynn *et al*., 2011; Grab *et al*., 2019). The relationship between biodiversity – including TR – and ecosystem functioning is often positive, although it is not necessarily strong or linear.

The total TR observed locally could also be relevant to ecosystem functioning occurring at larger temporal and spatial scales. If there are temporal fluctuations in the environment, higher TR at a site can reduce temporal fluctuations in functioning, resulting in greater stability *via* temporal insurance (Yachi & Loreau, 1999; Loreau & de Mazancourt, 2013). If TR varies across different local sites, among‐site dispersal can stabilize local ecosystem functioning by allowing species to track their optimal conditions, a biodiversity benefit known as spatial insurance (Loreau, Mouquet & Gonzalez, 2003; McGill, Sutton‐Grier & Wright, 2010). The loss of spatial and temporal insurance effects *via* the loss of biodiversity, especially phenotypic diversity, has been linked to lowered mean ecosystem functioning, greater variation in functioning, and lower stability (Schindler *et al*., 2010; Allan *et al*., 2011; Gross *et al*., 2013). The links between ecosystem processes such as the ones mentioned above at larger scales and benefits to people such as food production and protection against environmental hazards or climate regulation have been extensively documented (e.g. Millennium Ecosystem Assessment, 2005). Generally, the scale at which phenotypic diversity is measured is not necessarily the only scale at which it will affect functioning.

#### (b) *Conclusions and future directions*

The large literature on biodiversity–ecosystem studies suggests that higher biodiversity is usually – but not universally – linked to higher levels of ecosystem functioning and this relationship is often mediated by phenotypic diversity. However, studies about biodiversity and ecosystem functioning are heavily biased towards plant taxa (Balvanera *et al*., 2006; Cardinale *et al*., 2006), and the measured ecosystem functions are often limited to only a handful of common measures and traits, and use simplified systems (Hooper *et al*., 2005; Tilman *et al*., 2014). There is limited support that TR is a predictor of ecosystem function in other taxa as well (e.g. in animals; Gagic *et al*., 2015). The specific mechanisms and the strength of the relationship between TR and ecosystem functioning likely differ among ecosystems, traits, and functions.

The role of spatial scale is important. Most studies consider PD values at very large spatial scales [e.g. for entire clades, biogeographic regions, or large spatial grid cells; Jetz *et al*. (2014); Pollock *et al*. (2017); Strecker *et al*. (2011)], while ecosystem functions are typically measured and reported at much smaller (local) scales (Garnier, Navas & Grigulis, 2016). The relationship between TR measured for the entire species pool and for a local assemblage will vary, depending on how regional processes limit local diversity and how local processes non‐randomly select for species (Ricklefs, 1987; Shurin & Srivastava, 2005). There is empirical evidence that the loss of TR from the regional pool does reduce TR in local communities as well (Ernst, Linsenmair & Rödel, 2006; Smart *et al*., 2006; Flynn *et al*., 2009; Clavel, Julliard & Devictor, 2011), although the literature remains far from comprehensive. Even if TR at the regional scale directly affects TR at the local scale, it might not influence ecosystem functioning if there is high functional redundancy and/or if local processes strongly limit community diversity (Díaz & Cabido, 2001). Researchers have begun considering larger spatial scales and similarly identifying impacts of biodiversity on ecosystem functioning (Winfree *et al*., 2018; Zhang *et al*., 2018), but plot sizes remain relatively small (Zavaleta *et al*., 2010). Future studies need to ask how preserving phenotypic diversity at different scales (from global hotspots to local parks) might impact ecosystem functioning at local scales. If positive impacts are observed at increasing spatial scales, this would bolster the strength of the argument for considering evolutionary history for conservation *via* Link 2.

### (3) Is evolutionary history or phenotypic diversity associated with the human preference for novelty and variety?

#### (a) *Rationale*

Typically, arguments linking biodiversity to societal benefits explicitly consider benefits that are directly and measurably linked to ecosystem processes, such as soil fertility, fodder production and carbon sequestration resulting from productivity and nutrient cycling (Ehrlich & Wilson, 1991; Chapin *et al*., 2000). However, biodiversity is also the source of harder‐to‐quantify benefits associated with ‘human preferences’, including ideas and feelings of what is important, beautiful, or otherwise meaningful, all mediated by socio‐cultural, spiritual, philosophical, and/or historical factors (Díaz *et al*., 2015, 2018). An argument that humans prefer sets of species with increased PD or TR is not commonly made, but has merit given the importance of human subjectivity in societal choice, including those that drive conservation priorities (Vining & Tyler, 1999). Link 3 hypothesizes that humans may have preferences, through various mechanisms, for groups of species that contain more phenotypic diversity and/or more evolutionary history. This might occur if evolutionary history (measured by PD) or phenotypic diversity (measured by TR) captures values of interest to humans, for instance, by corresponding with the diversity of colours, structure and form (attracting varied and colourful birds at the feeder), or with very evolutionarily isolated species (lungfish as a zoo exhibition rather than a zebrafish). It also might occur if humans prefer natural variety in its own right (Tribot, Deter & Mouquet, 2018). However, very few papers explicitly test human preferences for PD or TR.

An argument has been made that humans have an evolved preference for nature (‘biophilia’), even especially for natural variety (which could potentially map onto phenotypic diversity) (McAlister & Pessemier, 1982; Simonson & Winer, 1992; Ratner & Kahn, 2002; Wilson, 2007). Arguments for biophilia tend to be couched in evolutionary psychology but have been criticized for their lack of specific mechanisms (e.g. Maier, 2012, pp. 222–227). Beyond such arguments, there are a few specific empirical studies of relationships between human preferences (whether aesthetic, cultural, or religious) and TR or PD. For example, there is some evidence that humans prefer groups of plants, both in gardens and in natural systems, that have higher TR (in terms of floral, foliage, and phenological traits) (Turpie & Joubert, 2004; Lindemann‐Matthies & Bose, 2007; Southon *et al*., 2017), although this is not always the case [see Knapp *et al*. (2012) for an example in which gardens have lower PD than natural sites]. Positive relationships between TR and human preference were found for Mediterranean reefs: surveyed individuals rated structurally diverse and colourful assemblages more aesthetic, and aesthetic appreciation was significantly correlated with both the TR and PD values for an assemblage (Tribot *et al*., 2016). Importantly, neither of these latter relationships was corrected for species richness, which was even more predictive of preference. In many cases though, human preference has strong taxonomic biases (Troudet *et al*., 2017): humans tend to value top predators (Clucas, McHugh & Caro, 2008), larger species, and mammals over birds or non‐avian reptiles [see references in Martin‐Lopez, Montes & Benayas (2007)] rather than explicit properties of sets such as PD or TR.

Interestingly, humans also often value rare objects, including rare natural entities such as uncommon bird species (Eklund & Hebert, 1997; Courchamp *et al*., 2006; Hall, Milner‐Gulland & Courchamp, 2008). This could possibly extend to species that are rare as the result of being evolutionarily or functionally distinctive. One motivator for public interest in conservation of species like the tuatara (*Sphenodon punctatus*) in New Zealand may be that they are both evolutionarily and morphologically distinctive as well as being endemic (Seabrook‐Davidson & Brunton, 2014).

#### (b) *Conclusions and future directions*

Most work examining connections between human preference and conservation was not designed directly to test evolutionary history or phenotypic diversity, and there is not enough evidence available to evaluate Link 3. Current studies are limited in number and are rarely designed to test directly whether evolutionary history or phenotypic diversity is linked to human preference directly, or *via* a sampling effect, whereby more diverse sets tend to include species we prefer. Future studies should consider testing human preference for PD or TR directly, for example asking ‘Do people favour national parks harbouring sets of distantly related species more than parks that are home to sets of closely related species?’ or ‘Do people search more frequently online for species with no close relatives and, if so, why?’ (Roll *et al*., 2016). We note that the potential variation in human values across cultures and world views regarding biodiversity may preclude any easy generalities.

### (4) Can evolutionary history and phenotypic diversity provide future options for humanity?

#### (a) *Rationale*

The previous arguments are focussed on the human‐centric benefits of evolutionary history that are realized more or less immediately (*via* Links 2 and 3). However, the value of evolutionary history could also be realized in the near or distant future. Link 4 captures the argument that protecting more evolutionary history (measured by PD), and thereby capturing more phenotypic diversity (measured by TR), will maintain greater future benefits for humanity, an outcome referred to as ‘option value’ (Faith, 1992, 2017).

Unfortunately, there are several versions of this argument that biodiversity maintains options for humanity. For example, some authors consider option value as including only the currently known uses for biodiversity (whether medicinal, agricultural, or spiritual), while others include unknown future uses that could arise at some further time point. Some authors assume that biodiversity will only be associated with future benefits, while others include the possibility of future detrimental impacts of biodiversity (Díaz *et al*., 2018). For example, Faith (1992, 2016, 2017) explicitly incorporates both known and unknown future contributions of biodiversity, and notes that given the rate of current planetary change, we cannot precisely define or predict which attributes of biodiversity we will value (and how much we will value them) at a future time. Maier (2012) specifically defines ‘option value’ as the benefit gained by maintaining elements of biodiversity (e.g. traits) in the absence of knowledge about their specific future benefits. The Millennium Ecosystem Assessment (2005) invokes ‘unexplored options for the future’, and recently the IPBES classification of nature's contributions to people includes reference to ‘yet‐to‐be‐discovered uses’ under the ‘maintenance of options’ category (Díaz *et al*., 2018). The validity of option value as an argument for evolutionary history likely depends on how it is defined and understood.

It is difficult to thoroughly evaluate whether evolutionary history helps to maintain options, given both the lack of agreement in the literature on the nature of option value, and the future‐focused nature of such options. A true test would require that biodiversity be measured initially, and then the benefits identified and tracked until some future time point. However, some general conclusions are possible. First, when the maintenance of options is used with reference to preserving only the known benefits of biodiversity at a future time period, these benefits will be realized *via* the same arguments and evidence presented for Links 2 and 3 and so those arguments should inform our evaluation. Second, where future uses of biodiversity are unknown, arguments that assume only positive future contributions of biodiversity without addressing the negatives should be suspect. If the future contributions of biodiversity are truly unknown, then the future contributions of evolutionary history and phenotypic diversity could be positive, zero, or negative (Maier, 2018). Díaz *et al*. (2018) assume both beneficial and detrimental values of biodiversity in this context: protecting evolutionary history might capture traits associated with invasiveness, competitiveness with crop species, or diseases, just as it might also capture sources of future medicines or foods. Any predictions for future contributions must include potential threats, but the attributes of biodiversity we will disvalue are also unknown, which weakens arguments that invoke unknown future options.

Arguments that assume net positive future benefits of evolutionary history (Faith, 1992, 2016) may be making an (unstated) assumption by treating the current benefits as an informative prior for the future benefits. The only explicit test of the predictive benefit of increased PD that we know is by Forest *et al*. (2007), who showed that across South African plants, subsets of species with higher PD were also more likely to come from genera with current value for human use. This pattern emerged because species with human uses tended to be phylogenetically clustered, but with different classes of use distributed in different genera, and a PD‐based sampling regime captured more genera overall. A second study offered consistent patterns: although PD was not evaluated directly, phylogenetic clustering of traditional medicines (i.e. across different ailments) was found for South African, Australian and New Zealand floras (20000 species in all; Ernst *et al*., 2016; Saslis‐Lagoudakis *et al*., 2012). If phenotypic diversity tends to contribute positively to beneficial uses currently, it seems likely that even in an uncertain future, TR or PD would be predicted to make positive, and potentially novel, contributions. The costs or negative impacts of biodiversity are less easy to quantify however.

#### (b) *Conclusions and future directions*

The maintenance of options is invoked in various forms in the evolutionary history and conservation literatures, and we suggest that the argument is supported only if (*i*) more phenotypic diversity predicts more current biodiversity benefits, and (*ii*) this transitively implies that phenotypic diversity will lead to more future benefits. Future research should both test, and, importantly, develop a prior probability for the actual value of future ‘unknown’ uses associated with TR and PD. Understanding the relative benefits and costs of maximizing the protection of PD and TR will remain difficult, however, given the time periods necessary over which to observe effects. There may be parallels in other fields for how best to consider unknown future values of things with and without strong priors on whether they are likely to offer benefit or harm.

### (5) Do greater evolutionary history and phenotypic diversity reduce species' extinction risk?

#### (a) *Rationale*

One goal for conservationists is to carry out actions that limit the loss of biodiversity from local to global scales (e.g. Aichi target 12 under the Strategic Plan for Biodiversity 2011–2020). Link 5 argues that prioritizing evolutionary history can facilitate this goal *via* its surrogacy for phenotypic diversity, if a group of species with higher evolutionary history also has a lower per‐species extinction risk (e.g. as compared to a randomly selected set of species of the same size). This argument may be relevant for multiple spatial scales, from whole assemblages to local communities: we discuss these separately [but see Keil *et al*. (2018) for discussion of extinction and spatial scales].

Traits provide the necessary link to extinction risk because species' traits mediate survival and performance under environmental stressors. There are clear links between specific species traits and extinction risk when evaluated at the scale of the entire phylogeny and for specific clades (McKinney, 1997; Orzechowski *et al*., 2015), although these interact with type of threat (Gonzalez‐Suarez, Gómez & Revilla, 2013; Orzechowski *et al*., 2015). In vertebrates, body size extremes (i.e. both large and small) are associated with high extinction risk (Cardillo *et al*., 2005; Liow *et al*., 2008; Ripple *et al*., 2017). Life‐history traits such as generation time can interact with spatial measures like geographic range size to affect species' extinction risk (Pearson *et al*., 2014). The argument linking TR and lowered extinction at the assemblage level is that by considering a set of species with higher TR we increase the probability of including at least some species that will survive in the face of unknown and/or changing stressors (Gonzalez‐Suarez *et al*., 2013; González‐Suárez & Revilla, 2013), i.e. a statistically mediated sampling effect. Capturing greater TR in a set of species may also preserve more species interactions such as mutualisms that prevent co‐extinctions (Rezende *et al*., 2007; Colwell, Dunn & Harris, 2012; Traveset, Tur & Eguíluz, 2017).

However, sampling‐effect arguments linking TR to decreased extinction should hold equally well in the opposite direction: maximizing TR also increases the chances of sampling species with traits associated with higher extinction risk. Two palaeontological studies are consistent with this alternative perspective: Liow (2007) found that species with longer fossil durations have average trait values, while Raia *et al*. (2016) found that specialization led to increased extinction of entire clades. If it is reasonable to assume that ‘average morphologies’ are groups of species that would contribute little to total TR, and specialization results in groups of species that contribute more to TR, these findings suggest a negative correlation between TR and *per capita* extinction rate. *Via* a sampling effect, it is likely that preserving greater phenotypic diversity has the potential to preserve key traits associated with extinction risk; unfortunately, without further research, it is not possible to determine the balance between those traits that increase *versus* decrease extinction risk for species.

An alternative mechanism for this link is that sets of species with higher PD might be more stable. In situations where clades are undergoing rapid radiations (e.g. cichlids and European whitefish), speciation may not be ‘complete’, and sudden environmental changes can actually reverse speciation *via* introgressive hybridization (Seehausen *et al*., 2008; Vonlathen *et al*., 2012). Sets of species with higher PD are also likely to be older because their terminal branches are, on average, longer (Steel *et al*., 2018). Increased reproductive isolation may be associated with the accumulation of trait differences occurring in the time since divergence. In these cases, higher PD may indirectly have the effect of selecting sets of older species that cannot undergo such reversals, decreasing the average extinction rate compared to a random set.

Unfortunately, we know of no direct empirical tests of the impact of prioritizing TR or PD on per‐species extinction risk at the regional scale and the indirect evidence is mixed. The loss of at‐risk species can lead to both the loss of more PD than expected at random [mammals and birds globally (Purvis *et al*., 2000); plants globally (Vamosi & Wilson, 2008)] or less than expected at random [amphibians globally (Greenberg & Mooers, 2017); plants in South Africa (Davies *et al*., 2011)]. Such patterns are the outcome of interactions between the heritability of extinction risk (*via* associated traits) and the relative contribution to PD by at‐risk species, and these vary by clade. For example, while extinction risk shows a mild phylogenetic signal in mammals (Fritz & Purvis, 2010), species that are highly evolutionarily distinct [which are expected to contribute disproportionately to total PD (Steel *et al*., 2018)] are not at heightened extinction risk (Verde Arregoita, Blomberg & Fisher, 2013). In amphibians, extinction risk is also heritable, but evolutionary distinctiveness (ED) and risk are (weakly) negatively correlated (Morelli & Møller, 2018), while in sharks and rays, ED and extinction risk are positively correlated (Stein *et al*., 2018). Without considering these additional contexts, it would be difficult to predict the expected relationship between evolutionary history, phenotypic diversity, and extinction risk.

At the smaller spatial scales relevant to ecological communities and ecosystems, preserving higher phenotypic diversity could reduce extinction risk by allowing for insurance effects. As discussed in Link 2, temporal insurance effects occur when higher TR in a local community provides a temporal buffer such that declines in sensitive populations are concurrent with growth of tolerant populations, stabilizing community dynamics and thus potentially reducing extinctions overall (Ives, Gross & Klug, 1999; Cottingham, Brown & Lennon, 2001).

#### (b) *Conclusions and future directions*

Support for Link 5 faces different challenges depending on the spatial scale of interest. There are well‐established relationships between particular traits and extinction risk at large scales, but there is no general mechanism linking sets of species with more phenotypic diversity to lower per‐species extinction, nor are there any empirical tests. Both are avenues for future research. Even at the community scale, we know of no experimental tests that link local TR and *per capita* local extinction rate [although many tests of the converse question show that extinctions tend to reduce local TR disproportionately (McKinney & Lockwood, 1999; Mouillot *et al*., 2013)]. Further, as with Link 2, conservation actions focussed on PD need to have effects relevant to the local scale. If conservation actions occur at larger scales, local‐scale TR may or may not be predictably affected.

### (6) Does evolutionary history or phenotypic diversity provide greater evolutionary potential?

#### (a) *Rationale*

The term ‘evolutionary potential’ originally referred to biotas having the potential for future evolutionary diversification (Brooks, Mayden & McLennan, 1992). Although current conservation policy is calibrated towards benefits in the present or very near future, humans are influencing the trajectory of biodiversity over much longer time scales. Myers & Knoll (2001) argued that humanity should take the long view and prioritize the Earth's future biota: such a focus might give priority to ‘hotspots’ of evolutionary potential such as regions of high neo‐endemism (Erwin, 1991; Brooks *et al*., 1992) with the hope that they will continue to generate biodiversity into the future. Link 6 argues that maximizing phenotypic diversity (Link 6a) or evolutionary history (Link 6b) will select assemblages with higher‐than‐average evolutionary potential.

Evolutionary potential (EP) is an ambiguous term in the conservation literature and has been used to describe various mechanisms by which some lineages or biotas persist, adapt, or diversify. We treat persistence as synonymous with reduced extinction risk (addressed in Link 5), and here discuss EP as either (*i*) the potential of lineages to adapt in response to future changes, and/or (*ii*) the potential for future diversification (e.g. the net production of new species). These involve very different mechanisms and imply different time horizons for conservation policy and so we discuss them separately.

##### (i) *Do sets of species with higher PD or TR have greater potential to adapt to future changes?*

There is widespread interest in determining the potential for lineages to adapt to global change (Baker *et al*., 2004; Aitken *et al*., 2008; Sunday *et al*., 2014; O'Brien *et al*., 2017). The question of whether sets of taxa encompassing high phenotypic diversity or evolutionary history are likely to differ systematically in terms of their potential for adaptation has received little theoretical attention, and most empirical tests can provide, at best, only indirect evidence. Many factors influence the ability of a species or population to adapt to future changes, including the amount of standing genetic variation (Lynch & Walsh, 1998; Bowen *et al*., 2016; Miraldo *et al*., 2016). This may indirectly relate to the PD in a set of taxa, since sets of species with higher PD are also generally ‘older’ sets of species, in the sense that their terminal branches are, in expectation, longer. If such species have more genetic variation, then PD will be indirectly correlated with adaptive potential. A few studies support this: in birds, evolutionarily isolated species do tend to have greater phylogeographic structure (Smith *et al*., 2017) and a (slightly) higher number of subspecies (Phillimore *et al*., 2007). Mammal species in the tropics tend to be both more evolutionarily isolated and contain more genetic diversity than mammal species in temperate regions (Miraldo *et al*., 2016). The generality of these patterns is not yet established, but these limited results suggest that species that contribute disproportionately to PD might also be species with high genetic variation and thus adaptive potential.

Even fewer studies have looked for a relationship between phenotypic diversity (TR) in a group of taxa and their adaptive potential. One potential mechanism is through a sampling effect, if a set of species with greater TR has a greater chance of including key traits necessary for successful adaptation and persistence. Again the alternative pattern is possible, and high TR could lead to a greater chance of including traits that would render lineages less adaptable. Microbial experiments confirm that the presence of particular trait values can increase the likelihood that a community adapts to changing and increasingly unfavourable conditions (Carlson, Cunningham & Westley, 2014; Low‐Décarie *et al*., 2015). Microbial systems in general should be amenable to testing the link between adaptability and evolutionary history or phenotypic diversity. Existing evidence is too limited to cohesively link TR and adaptive potential.

##### (ii) *Do sets with higher PD or TR have greater potential for future speciation?*

Geographic regions with fewer closely related lineages often have higher per‐lineage speciation rates – such that clades and regions that are currently species poor are likely to be hotspots of ongoing and future speciation (Rabosky, 2009; Schluter & Pennell, 2017; Rabosky *et al*., 2018). There is compelling evidence from both fossil and phylogenetic data that the presence of similar (or closely related) species in an environment inhibits diversification [see Schluter & Pennell (2017) and references within]. If this were a key regulator of diversification, then selecting sets of species to maximize PD in a geographic region might facilitate future speciation by limiting the relatedness of protected species. On the other hand, predicting future diversification is generally fraught (Ezard *et al*., 2011) and a recent explicit test of the relationship between PD and evolutionary potential measured as total lineages through time found no evidence for PD as a predictor of subsequent biodiversity (Cantalapiedra et al., in press).

Speciation rate may also be affected by species' attributes or traits (Mitter, Farrell & Wiegmann, 1988; Jablonski, 2008; Goldberg *et al*., 2010). If traits influencing speciation are passed on to daughter lineages, high speciation rate itself would have a phylogenetic signal. The highly unbalanced shapes of published phylogenetic trees (Mooers & Heard, 1997) is consistent with this pattern of heritability. This would also imply that species in depauperate parts of the tree of life have characteristics that give them lower‐than‐average speciation rates, in essence making them ‘dead clades walking’ (Jablonski, 2002). If we assume that these historical relationships between traits and speciation rates will hold in the future, then conserving evolutionarily isolated species with few close relatives (as happens when maximizing PD) would actually lower average evolutionary potential compared to a random choice (Erwin, 1991; Jablonski, 2002). A recent analysis using fossil trees for multiple clades found that prioritizing diversifying lineages protected more total lineages (their estimate of evolutionary potential) through time than did random choice, although gains were modest (Cantalapiedra et al., in press). If speciation rates typically have a phylogenetic signal, high PD sets of species will preferentially capture ‘dead clades walking’ and would not act as a surrogate for future diversification.

#### (b) *Conclusions and future directions*

Tests of Link 6 must consider time periods long enough for adaptation and diversification to be observed, making them difficult. Existing evidence is insufficient to link evolutionary history or phenotypic diversity to evolutionary potential. Further, evolutionary potential is rarely evaluated in the context of conservation since the time scales involved are rarely relevant to current conservation activities. Thus, protecting evolutionary history to foster evolutionary potential is a poorly supported argument and at odds with most conservation programs. That said, more work is required to answer outstanding questions: do sets of taxa chosen to maximize PD differ systematically in their genetic diversity and ability to adapt? Does the fossil record contain signals linking standing PD and subsequent diversity (see, e.g. Huang, Goldberg & Roy, 2015)? Finally, can we use experimental microcosms to track diversification and identify relationships between past and future diversity (Bell, 2013; Low‐Décarie *et al*., 2015)?

## VI. ASSESSING THE CONSERVATION VALUE OF EVOLUTIONARY HISTORY

Uncertainty in our understanding of the structure and function of natural systems creates major challenges in carrying out effective and efficient conservation decisions. The rapid rate of modern biodiversity loss only compounds the problem. If a single conservation target could be associated with multiple values – decreased extinction rates, increased future adaptation, maintenance of beneficial future options, and protecting ecosystem functioning – it would prove a very useful tool in the face of such uncertainty. The phylogenetic gambit suggests that if prioritizing evolutionary history captures variation in form and function that benefits people, then evolutionary history could be such a tool. Given the paucity of explicit theoretical or empirical assessment of this gambit, we here provide the most comprehensive analysis of these claims to date. While there is support for the conservation value of evolutionary history, this support varies widely among arguments: some links are poorly supported, some have strong support, and for some links, there is still too little evidence to draw a conclusion (Table 2).

Most of the arguments for conserving evolutionary history in Fig. 1 rely on a surrogate relationship between evolutionary history and phenotypic diversity, and so identifying support for this relationship is essential. There is good theoretical and empirical support for a surrogacy relationship between TR and PD, and preserving greater PD usually leads to greater average TR compared to random. At least under commonly applied models of evolution and for empirical analyses at large spatial scales, Link 1 appears to be a reasonable assumption.

The arguments receiving the strongest empirical and theoretical support were those relevant at small spatial scales (local to regional) and over short (current) time scales. In particular, a number of studies have found good (although variable) support for the value of PD (*via* TR) for increased ecosystem functioning (Link 2) and stability of functioning resulting from temporal and spatial insurance and sampling effects (Links 2 and 5). While the literature suggests that greater phenotypic diversity is associated with increased ecosystem functioning, the linkage may at times be complex, context dependent, or system specific, and further work focused on the relationship between TR and functioning is necessary. Phenotypic diversity may also affect local extinction rates, if sampling effects and temporal complementarity moderate fluctuations in populations, and/or prevent co‐extinctions.

The key limitation to some arguments is that the scale at which conservation activities and plans based on evolutionary history tends to occur (regional, clade) is not that at which the benefits of interest are quantified (local). The question of how the relationship between evolutionary history or phenotypic diversity and ecosystem functioning changes as they are aggregated over larger spatial and temporal scales is a timely one and beginning to receive attention in the literature. For Links 2 and 5 to hold, conservation activities focused regionally should as a result also prevent the loss of diversity at the local scale (see Vellend *et al*., 2017) or protect ecosystem functions across multiple sites (such as by maximizing beta‐diversity in TR). Further research is essential to understand the theoretical expectations and empirical patterns of relationships between evolutionary history and phenotypic diversity across scales.

**Figure 3:**
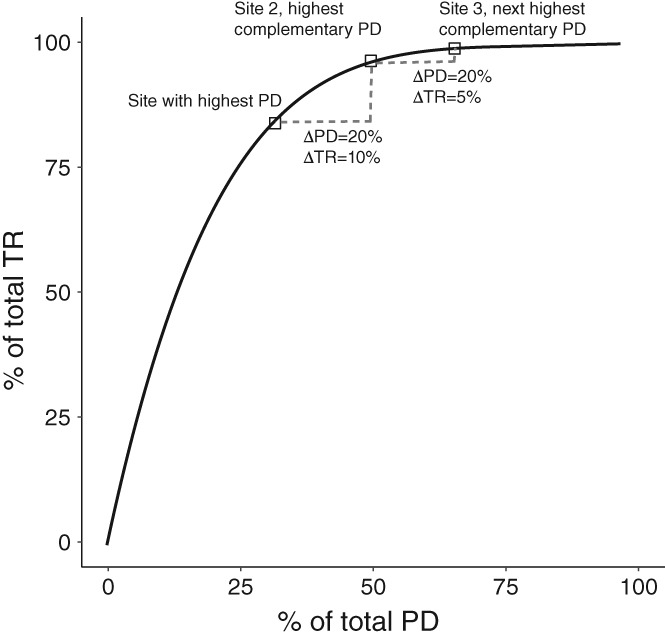
The impact of complementarity‐based spatial prioritization on the strength of Link 1 (see Fig. 1). In general, additional sites chosen to capture complementary phylogenetic diversity (PD) – relative to those sites chosen previously – should also capture more trait richness (TR) and increase the strength of the PD–TR linkage. However, the relative impact of prioritization depends on the position of those sites along the surrogacy curve (and the shape of the curve) – prioritization will be most effective where the slope is steep, and less relevant as the curve approaches saturation.

The issue of spatial scale is also critical for many conservation‐prioritization approaches and provides a useful connection between the relationships in Fig. 1 and more‐applied conservation activities. Conservation plans that maximize complementary in PD, for example, iteratively select sites so as maximally to incorporate additional evolutionary history and increase the total PD captured with each new site (Carvalho *et al*., 2017; Cadotte & Tucker, 2018). The impact of spatial complementarity here is to move up along a surrogacy curve relating PD to other values of conservation interest. For example, in the case of the surrogacy curve of PD for TR, accumulating sites to add greater PD should generally increase the TR captured (Fig. 3), at least until the point at which the PD–TR relationship saturates (Mazel *et al*., 2018). This impact of spatial prioritization may have a strong impact, unpredictable impacts, or no impact on the arguments in Fig. 1. It may may lead to a stronger correlation between PD and TR, at least when the PD‐TR surrogacy curve is steep such that complementary PD should result in the accumulation of additional TR as well (Fig. 3). Links 2 and 4, that also rely on Link 1, might therefore also benefit from spatial prioritization of PD. For Links 5 and 6, for which it is uncertain what the balance of negative impacts *versus* positive impacts of biodiversity might be, spatial prioritization could have negative or positive impacts. Scaling of surrogacy across spatial scales is a clear area for further focused work.

Caution is required when arguing that evolutionary history will yield greater benefits through increased human preference (Link 3), lower global extinction rates (Link 5), or higher evolutionary potential (Link 6). While reasonable and logical arguments can be constructed for these three links, we found very few tests, and what evidence we did identify was indirect and generally not from conservation‐focussed studies. For Links 4–6, their longer temporal and/or spatial scales have made them difficult to test. In addition, arguments about conservation values that rely on time scales of millions of years (such as evolutionary potential) are not likely to inform mainstream conservation activities (Barraclough & Davies, 2005; Purvis *et al*., 2005). Thus, these links should not be central in driving the use of evolutionary history for informing conservation decisions, at least in the absence of more directed research attention that produces positive support.

## VII. CONCLUSIONS

(1) Our knowledge of the mechanisms underlying the relationship between evolutionary history and human‐centred conservation benefits is imperfect, often indirect, and incomplete. Surprisingly, even after 25 years of research, the strength of evidence linking evolutionary history to human‐centric benefits is still fragile.

(2) Biologists tend to hold the general expectation that more biodiversity leads to a net positive benefit to people, even though there are some added risks (e.g. disease). If this expectation is justified, we might expect positive benefits of evolutionary history and trait diversity *via* sampling effects or temporal insurance, even into the more distant future.

(3) Notwithstanding, it will be important for future work to fill in knowledge gaps through direct tests of the arguments we define here. Until there are stronger empirical tests, evolutionary history should be treated as complementary to existing information rather than as a focal measure, at least where the goal is specific human‐centric benefits.

(4) Even if detailed information regarding the relationships between PD and ecological processes and future uses is unknown, optimizing the total evolutionary history protected should increase the chance of sampling important differences among species and useful features. Again this requires that we assume there is a net positive benefit of evolutionary history (see Section V.4 for more details). Evolutionary history in this role can contribute to conservation, as a tool to buffer against our limited understanding of the relationship between ecological form and functioning, and our limited understanding of future uses for biodiversity. As an additional layer of information, for example, evolutionary history could inform the evaluation of the success of existing protected areas, or the effectiveness of focussed conservation programs such as seed banks.

(5) We reiterate that our evaluation of the evidence did not consider any intrinsic value arguments for preserving biodiversity. We suggest that more work be directed at linking evolutionary history with a wider range of values (see e.g. Chan, Gould & Pascual, 2018)

## IX. AUTHOR CONTRIBUTIONS

All authors contributed substantially to the literature searches and subsequent framing of this review, with different authors leading the writing of specific subsections. C.M.T. and A.O.M. edited, coordinated, and cajoled.
